# Dietary behaviours of young South Asians in Australia: insights from a qualitative study

**DOI:** 10.1017/S1368980025101250

**Published:** 2025-10-16

**Authors:** Aboli Pawar, Amani Fuad, Priya Iyer

**Affiliations:** The University of Sydney, Nutrition and Dietetics Group, Susan Wakil School of Nursing and Midwifery, The Charles Perkins Centre, Sydney 2006, Australia

**Keywords:** South Asians, Dietary behaviours, Acculturation, Food choices, Eating practices

## Abstract

**Objectives:**

To describe the drivers of dietary behaviours among young South Asian adults (18–35 years) in Australia and examine the influence of acculturation and the host environment in shaping these behaviours.

**Design:**

A cross-sectional qualitative study design was used to collect data through Zoom focus groups. The analysis of the qualitative data followed Vears and Gilliam’s inductive content analysis approach. Demographic data were presented descriptively, and the findings were reported in accordance with the standard for reporting qualitative research.

**Setting:**

Sydney, New South Wales, Australia.

**Participants:**

A total of twenty-one young South Asian migrants (aged 18–35 years), mostly from India and Pakistan, participated in four online focus groups.

**Results:**

Six main themes were identified, highlighting the complex interplay of acculturation, cultural identity, family influence and other socio-economic factors in shaping dietary behaviours. (1) Acculturation and exposure to diverse cuisines, (2) interplay between religion and culture during special occasions, (3) influence of social networks and community norms, (4) structural and practical constraints shaping food choices, (5) shifting perceptions around healthy eating and (6) empowerment through knowledge. Participants reported incorporating Western and multicultural cuisines, often balancing convenience and cultural preferences.

**Conclusions:**

Numerous drivers, including but not limited to acculturation and the host environment, influence the dietary behaviours of young South Asian adults in Australia. Despite limitations in representativeness, the study provides valuable insights highlighting the need for inclusive health promotion efforts for Australia’s growing South Asian population.

Migration from South Asia (India, Sri Lanka, Nepal, Afghanistan, Pakistan, Bangladesh, Bhutan and Maldives) has seen a steep growth in Australia, with India and Nepal being among the top source countries from 2012 to 2022^(1)^. The Australian Bureau of Statistics predicts the Indian-born population to reach 1·07 million by 2035 and 1·4 million by 2045^(2)^. South Asian migrants (SAM) predominantly follow skilled migration pathways, resulting in a younger migrant demographic, largely aged between 20 and 39 years^(3)^. This life stage is often critical for establishing dietary habits and lifestyle behaviours that influence long-term health outcomes^(4)^. Additionally, with evidence of increased acculturation reported in those who migrate at an younger age, this age group reference is more relevant^(5)^.

Compared with non-migrant populations, SAM are at a higher risk of developing Type 2 Diabetes (T2D), with some studies reporting incidence rates of 57 % *v* 31 %^(6,7)^. Similar disparities have been reported in other high-income countries such as Canada with higher rates of T2D and hypertension seen in this population^(8)^. Although some SAM may initially benefit from the ‘healthy migrant effect’, whereby newly arrived migrants exhibit better health than native-born population, this advantage diminishes over time^(9)^. This decline is frequently associated with acculturation, a process by which migrants adopt the cultural norms, values and practices of the host country^(10)^.

Acculturation is a multifaceted concept encompassing behavioural, psychological and dietary adaptation and has been linked to shifts in food choices, meal patterns and physical activity levels^(11)^. Among SAM, the transition to Western dietary patterns, characterised by increased intake of ultra-processed foods and decreased consumption of traditional staples, has been associated with greater risk of obesity and related non-communicable diseases (NCD)^(11)^. Poor diet quality with higher energy density has been reported in SAM in high resource countries^(12)^. The impact of time since migration and environmental factors such as food accessibility, sociocultural expectations and identity negotiation further shape these dietary behaviours^(13)^. However, despite these reported risks, the dietary behaviours of SAM in Australia are poorly understood, particularly among young adults navigating dual cultural influences.

Understanding food culture is essential to contextualising dietary behaviours and practices within SAM. Although South Asian countries are geographically grouped, the food traditions, religious practices and dietary customs are diverse and varied^(14)^. Nonetheless, there are overarching similarities such as the use of spices, grain-based staples and plant-based diets that provide some rationale for grouping these populations in studies of dietary acculturation. Still, distinctions must be acknowledged, for example, Afghanistan’s food culture is influenced by other bordering countries in Central Asia and the Middle East and the Maldives with Southeast Asia, potentially limiting the homogeneity of the broader South Asian classification^(15)^. Furthermore, dietary patterns have been impacted by political factors in some of these countries^(16)^. Additionally, the diversity in culture has also been highlighted within individual South Asian countries such as India^(17)^. Despite these variations, a critical examination of such groupings is essential to accurately interpret findings and inform culturally responsive interventions.

Given the growing population of SAM in Australia, the elevated burden of diet-related NCD and the limited evidence on dietary behaviours within this cohort, there is an urgent need for focussed research. Therefore, this study aimed to explore the factors driving dietary behaviours among young SAM in Australia, with specific attention to the role of acculturation and environment influences. A qualitative approach using focus groups was chosen as the most appropriate method to explore the complexity of experiences and perspectives in this population^(18)^.

## Methods

### Study setting

Utilising an exploratory, descriptive qualitative design with focus groups and guided by a constructivism philosophical paradigm^(19)^, this study was conducted in Sydney, New South Wales, Australia, with appropriate ethical approval (2024/145). Constructivist paradigm^(19)^ that assumes that knowledge and meaning are socially constructed was appropriate for exploring dietary behaviours that are shaped by complex multifaceted contexts, which vary from person to person. Influenced by this approach, focus groups were selected as the most appropriate method to explore shared cultural experiences and social dynamics influencing dietary behaviours, which may be less visible in one-on-one interviews. This approach facilitated interaction and deeper insights through group discussion, particularly useful when exploring food practices and acculturation^(20)^. The findings are reported according to the Standards for Reporting Qualitative Research^(21)^. Given the qualitative descriptive design with a constructivist approach^(19)^, sample size was not pre-established, instead data saturation guided data collection. Data saturation was assessed through iterative review of themes after each focus group.

### Participants and recruitment

Utilising a combination of purposive^(22)^ and snowball^(23)^ sampling, young adults (18–35 years) who self-identified as SAM (Afghanistan, Bangladesh, Bhutan, India, Maldives, Nepal, Pakistan and Sri Lanka) were recruited using flyers circulated through social media platforms, cultural associations and friends of friends’ networks.

Participants were eligible if they were young adults aged 18–35 years migrated from South Asia, who self-reported as either (see online supplementary material, Supplemental 1):

-First-generation migrants living in Australia for at least 1 year.

-Second-generation migrants (born in the host country to first-generation migrant/s with at least one parent meeting this criteria).

-Able to communicate in English language

Both first- and second-generation SAM were included to explore a broader range of acculturative experiences. Intergenerational differences have been reported in multiple facets among this group^(24)^. Inclusion of both groups enabled the study to capture these generational nuances. Screening and informed consent were managed via secure Research Electronic Data Capture (REDCap) platform^(25,26)^.

### Data collection

**Figure 1.**
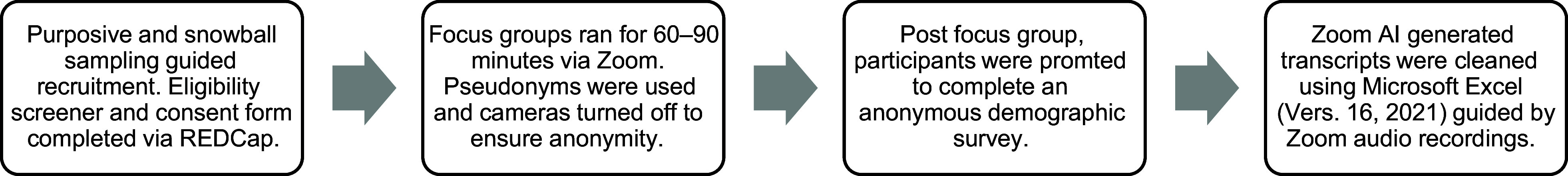
Study procedure.

Four focus groups using a semi-structured guide^(27)^ (see online supplementary material, Supplemental 2), was designed by the primary researcher (PI) with input from co-researchers (AF and AP), based on literature examining acculturation^(28)^, dietary behaviours among SAM and research aims. The first focus group was used to pilot these questions to enable refining for future sessions, although no further revisions were indicated. Focus groups comprising of four to seven participants (Mean 5·25 ± 1·26) lasting for a mean duration of 92·5 min, were conducted in English by two researchers (AP and AF) using Zoom (Figure 1). Zoom was considered for ease of participation and supported by the documented benefits of online focus groups in cross-cultural studies^(29)^. To ensure robust discussions and contextual exploration, researchers AP and AF received prior training. The training assisted moderators to manage online group dynamics, use participant pseudo-names regularly and provide space for participants to share information. Participants were encouraged to take turns and the moderators checked in with each participant to promote inclusive participation. AP (India), AF (Pakistan) and PI (India) identify as first-generation SAM and consciously used reflexivity throughout, consistent with the constructivist paradigm in the research^(30)^. Reflexivity was actively maintained to acknowledge and critically examine the researchers’ own backgrounds, beliefs and potential biases that could influence data collection and interpretation. Given that all the researchers shared similar cultural and migration experiences with many first-generation participants, this positionality offered both opportunities and challenges. While it facilitated rapport-building and deeper insights during focus groups, it required careful self-awareness to avoid assumptions or over-interpretations. Maintaining reflexivity helped the researchers to remain open to diverse narratives that diverge from own experiences. One researcher facilitated each focus group while another observed and took field notes taking turns for different focus groups, maintaining positionality in qualitative research. The audio recordings and the Zoom AI-generated transcripts were de-identified, checked using the audio recording and cleaned using Microsoft Excel (Vers.16, 2021) by one researcher, while the other cross-checked the work by alternating responsibility for each focus group. A detailed audit trail was maintained throughout the process. NVivo (Ver 14)^(31)^ enabled data management for qualitative analysis. Participants completed a short demographics survey via REDCap after the focus group (see online supplementary material, Supplemental 3). The survey collected information about their dietary preferences, gender, anthropometry, occupation, living arrangement and marital status. Self-reported height and weight were used to calculate the body mass index (BMI) using the criteria outlined by the World Health Organisation^(32)^. These were included to contextualise findings.

### Data analysis

Guided by Vears’ and Gillam’s inductive content analysis approach for qualitative synthesis^(33)^, data were read, understood, coded and recoded by two researchers (AF and AP), with any disagreements resolved by a third researcher (PI). The transcript was read, familiarised and the first-round coding was initiated, with big picture meaning units being identified during coding. Subcategories and fine-grained codes were identified through second round coding, which were further refined allowing for better synthesis and interpretation of results^(33)^. An inductive approach allowed themes to emerge from the data without being constrained by predefined theoretical models. While acculturation and dietary behaviours were central to the study aims, they were explored through participants’ narratives rather than being guided by a specific acculturation framework. This approach was chosen to allow a more grounded understanding of participants lived experiences. Data analysis occurred simultaneously with data collection until data saturation was achieved. Whilst data saturation was achieved (no new themes generated) with the third focus group, the fourth focus group proceeded as scheduled due to confirmed participants. Participants were grouped based on availability and age but not explicitly by cultural subgroup or generational status due to the exploratory nature of the study. However, demographic information including generational status was collected to allow contextual interpretation. Participant characteristics were reported using descriptive statistics derived using IBM SPSS Statistics v. 29 (IBM Corp. Released 2023. IBM SPSS Statistics for Windows, Version 29.0.2.0: IBM Corp).

## Results

A total of twenty-one participants aged between 18 and 35 years, from India (*n* 11), Pakistan (*n* 6), Bangladesh (*n* 2) and Sri Lanka (*n* 2) participated in four online focus groups. While eight countries were initially included in the eligibility criteria, the final sample represented only four, with 81 % from India and Pakistan. Comprising of 56 % female and 44 % male, majority were single (*n* 13), living with family (*n* 16). Participants self-reported a mean weight of 72 kg ±18·6 and a BMI of 24 ± 4·5 (Table 1). Of the twenty-one participants who took part in the focus groups, only sixteen completed the demographic survey in full, although all participants provided valuable qualitative data through focus groups. As a result, some demographic information has smaller sample size (*n* 16), details of which are footnoted in Table 1.

**Table 1.** Characteristics of participants

Participant characteristics	*n*	%	
Country of origin (*n* 21)			
India	11	52	
Pakistan	6	28	
Bangladesh	2	10	
Sri Lanka	2	10	
First-generation SAM* 11 52 Second-generation SAM* 10 48			
Other demographic characteristics (*n* 16)			
Gender			
Female	9	56	
Male	7	44	
Occupation			
Full-time worker	5	31	
Student	11	69	
Dietary practices			
Halal	10	63	
Vegetarian (milk only or milk and egg or no egg)	4	25	
Other	2	13	
Marital status			
Married	3	19	
Single	13	81	
Living arrangement			
Lives with family	16	100	
Anthropometry	*n*	Mean	sd
Height (cm)	16	170	12
Weight (kg)	15	72	19
Body Mass Index	15	24	5

Some demographic data were missing from five participants. Percentages are calculated based on the data available for each variable. *South Asian Migrants.

Notably, 100 % (*n* 16) of participants completing the survey reported living with family, which is particularly relevant in interpreting findings related to decision-making regarding dietary practices, food access and familial influence. Particularly important as several participants indicated that food choices were often shaped by the preferences and practices of other household members, especially parents.

**Figure 2.**
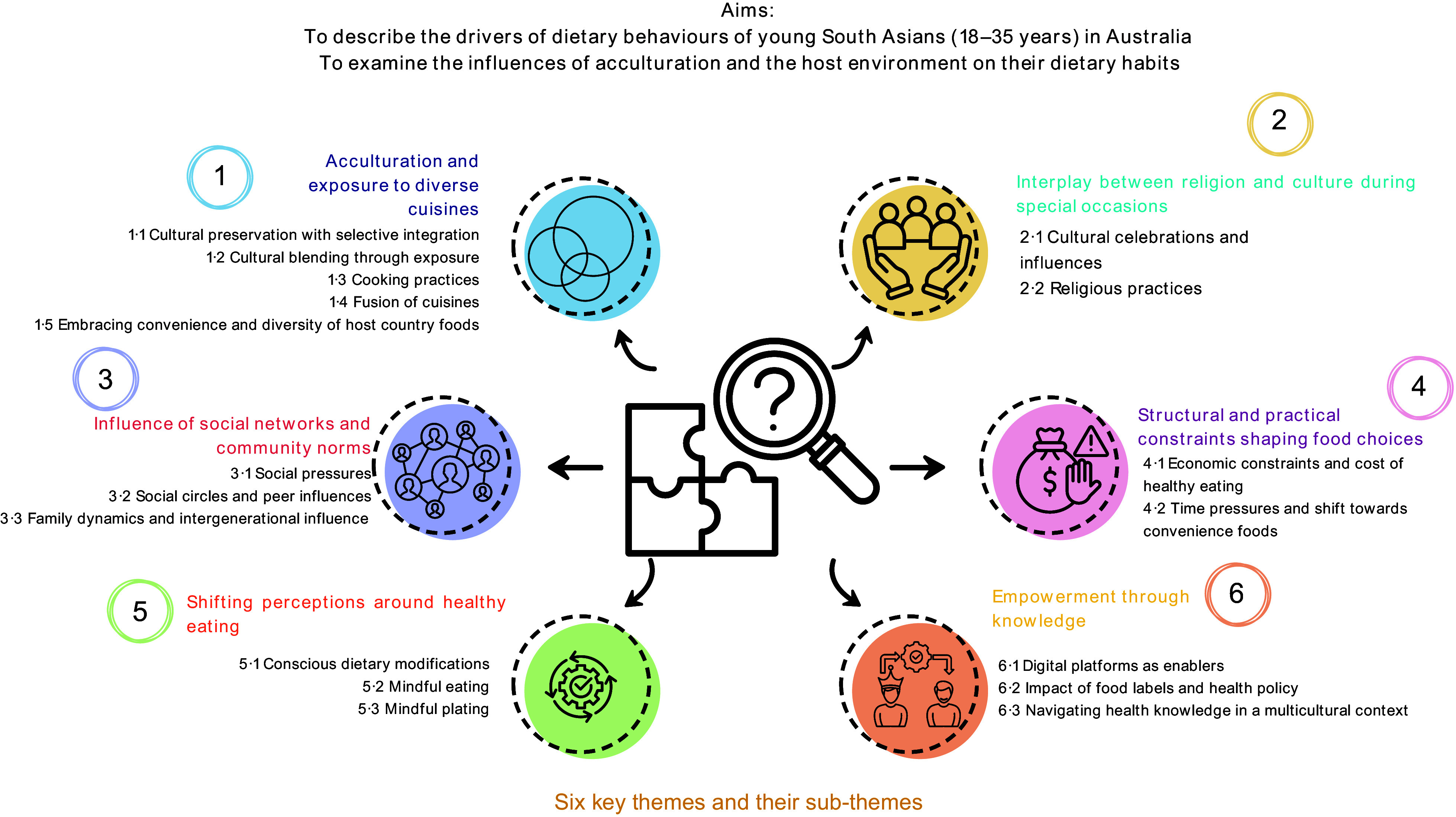
Key themes.

Inductive analysis identified six key themes, representing various factors such as acculturation, environment and culture influenced the dietary behaviours of young SAM (Figure 2).

### Theme 1: Acculturation and exposure to diverse cuisines

Participants identified the influence of acculturation in their eating practices. They highlighted how exposure to wider cuisines outside their cultural norms was introduced by engaging with the broader society. Specific components are described within these two related sub-themes.

#### Sub-theme 1·1: Cultural preservation with selective integration

Whilst participants recognised preserving their South Asian traditions, they identified themselves as being open to trying novel cuisines from their host country. Many participants indicated retaining their traditional practices at home, while opting for Western or other international cuisine when eating out socially.

#### Sub-theme 1·2: Cultural blending through exposure

Participants described their unique experiences navigating and integrating various cultural cuisines into their eating patterns. They reported their home diets as being rooted in traditional South Asian cuisine but influenced by multiculturalism, leading to a blend of Indian, Pakistani, Mediterranean, Italian and East Asian foods.

*I normally have food at home…I have been born and raised in Australia, and even though my parents are from India, and half of our dishes are Indian or Pakistani…We lived in the eastern suburbs…We have, like Italian dishes and Chinese dishes…[we] like experimenting with Asian cultures and Italian cultures for food as well as Lebanese. So, we are kind of more mixed in that point. As for cultural celebrations, we tend to either have Indian food or Mediterranean food* {Focus Group 4; Participant 6, India}

*…like 90 % of the food is influenced by [my]culture because my mom is cooking, and that’s the food she’s most comfortable cooking…though sometimes there is a bit of fusion…if I’m going to eat out with friends…it’s a lot of the non-Asian foods…* {Focus Group 1; Participant 1, India}

*So, rice was a staple, and I think it’s still a staple today[and]I would say that we do incorporate foods from like other cultures like we try Mexican…Italian, Lebanese. Yeah, all of those.* {Focus Group 1; Participant 2, Sri Lanka}

Participants further highlighted modifying their cooking methods to integrate both traditional South Asian foods and Western cooking practices.

#### Sub-theme 1·3: Cooking practices

Participants discussed incorporating cooking techniques such as air frying, noting that this method was not commonly used in their home countries. They also mentioned adopting ‘healthier’ cooking practices such as steaming, grilling and baking. Additionally, they highlighted a reduction in oil usage during cooking and an increased inclusion of fresh salads that require no cooking as new changes.

*…we use an air fryer a lot, which is something…that isn’t used back home …* {Focus Group 1; Participant 2, Sri Lanka}

*…parents have started using less oils. [We] started having salads more often here….* {Focus Group 2; Participant 7, India}

#### Sub-theme 1·4: Fusion of cuisines

Participants reported a shift in their cooking habits where South Asian flavours were infused into Western meals such as ‘pasta’, reflecting a culinary merging shaped by availability and new lifestyle.

*…my mum cooks like a lot of Desi food [and] on the odd occasion, she cooks…vegetarian pasta…* {Focus Group 2; Participant 5, Pakistan}

#### Sub-theme 1·5: Embracing convenience and diversity of host country foods

Participants expressed a preference for Western foods reportedly due to their reduced preparation time, suggesting a shift away from their traditional diets. While they noted to be cooking and eating more Western cuisines, they acknowledged the influence of family preferences. Enjoying a variety of cuisines from the multicultural host country including ‘Greek’ and ‘Mediterranean’ was however echoed.

*…I can only cook Western meals…and find myself eating Western meals…I don’t really find myself getting South Asian takeaway if I’m alone, so I usually tend to eat Western food…* {Focus Group 3; Participant 3, India}

*…being in a community that’s so multicultural… you get exposure to…different cuisines, but [they] still cater to your dietary requirements, like most of the things in Western Sydney, for instance are Halal…things like Middle Eastern food or just pizza shops and burger shops in general is not something that would traditionally be a South Asian thing, but because it’s readily available and it caters to…dietary preferences. It’s something that has increased in our diet…* {Focus Group 1; Participant 1, India}

### Theme 2: Interplay between religion and culture during special occasions

Some participants outlined how religion and culture are intertwined and influenced food choices. It was noted that while cultural celebrations allow wider celebratory food choices, religious dietary restrictions still informed food choices.

#### Sub-theme 2·1: Cultural celebrations and influences

Participants communicated that culture was a profound driver of their dietary behaviours. Particularly, during festivities and celebrations like Eid, Diwali and weddings, South Asian dishes were relished and preferred acknowledging that it is acceptable and common to be ‘indulged in more “unhealthy” foods’.

*…being in the South Asian community, especially around festival times in particular…things like events, parties just being part of the broader South Asian diaspora does definitely influence things that I eat. I think, typically on those festival days, or party days…you’ll eat food that are probably a bit less healthy, and you’ll definitely usually be eating South Asian food, at least.* {Focus Group 3; Participant 4, India}

#### Sub-theme 2·2: Religious practices

Participants mirrored the religious diversity within South Asia. They identified that certain foods, or dietary habits being regarded as important and pertinent to specific religious events. A common factor shared between these religions was the periodic fasting although the requirements varied across religions (abstinence of all food and fluids to selective items).

*When it comes to religious celebrations, where I have to be vegetarian, I have to fast longer especially during puja’s…I can’t have any meat or fish during any religious celebration.* {Focus Group 1; Participant 5, Bangladesh}

*Ramadan…is predominantly…fasting month…our eating schedules…aligns with what is expected of us during this fasting period. So, for example, whilst outside of this time, I would be having breakfast, perhaps in the late morning. It involves changing that timing to much earlier. Same with when it’s the second time I get to eat, so instead of around noon time, this falls around sunset. But then also with timings aside, there is also an increased consumption of dates and water, and a reduction of having really fat heavy foods in order to essentially keep us going for longer given we only have a few hours within the day itself that we allow ourselves to eat* {Focus Group 1; Participant 3, India}

### Theme 3: Influence of social networks and community norms

Inductive analysis revealed several social influences of food choices among young SAM. Participants recognised the challenges of navigating the multifaceted landscape of social and economic factors, personal preferences and familial expectations all of which influenced their dietary choices

#### Sub-theme 3·1: Social pressures

Participants mentioned that social pressures within South Asian communities, such as ‘judgmental attitudes toward body weight’, individual’s food preferences and choices impacted their food intake, with participants reporting to be eating larger meal portions. This was exemplified by anecdotes shared by participants about their experiences visiting community members’ houses or during community events, where eating large meals and big portions signified gratitude and respect.

*…the just a social pressure example I can say that usually whenever we’re like visiting someone, and then we’re having dinner with them or anything. They usually just pressure you into eating more, even if you’re on diet…they kinda pressure you a lot into eating more food, even if you’re full. There is that one social pressure.* {Focus Group 3; Participant 3, India}

#### Sub-theme 3·2: Social circles and peer influences

It was found that the social circle had a distinct impact on what foods were being eaten in social settings. Some participants preferred to eat South Asian foods when with friends, while others reported the opposite.

*…being part of the South Asian community does influence in a way that when we’re meeting more of our fellow community members…you tend to have those similar cuisines that you have back home…* {Focus Group 3; Participant 2, Pakistan}

#### Sub-theme 3·3: Family dynamics and intergenerational influence

Participants reported that family structure and dynamics greatly influenced their food choices, stemming through their upbringing from childhood and/or conscious choices of take away foods to suit family preferences.

*[I] don’t have a strict dietary preference, but in terms of seafood I feel like cause I’m [from a South Asian region], which is…landlocked…And typically in our culture we don’t consume seafood cause we don’t…have access to the sea. So, I feel like inadvertently just growing up with my parents, not eating that kind of stuff. It just like passed on to me. And so, when I would like go out and kinda like see seafood on the menu, it’s just like a natural like aversion, just because I’ve never really had it before.* {Focus Group 3; Participant 1, India}

*…so, choosing takeaway, then I’ve always picked from a Western outlet. But if my mom also wants [takeaway], then I’ll obviously pick from a South Asian place…* {Focus Group 3; Participant 1, India}

### Theme 4: Structural and practical constraints shaping food choices

#### Sub-theme 4·1: Economic constraints and cost of healthy eating

Monetary factors influenced dietary choices as participants stated that more expensive and oftentimes healthier food options may be dismissed to save money. Also, it was found that takeaway food was preferred if finance was a non-issue.

*One major thing that kind of makes it harder to eat healthy here is the…high prices for food…inflation is a very big factor…eating out eating healthy at a restaurant is much more expensive now, and even if you want to cook healthy It’s more expensive than it was before* {Focus Group 3; Participant 3, India}

*…If money is not a problem, then I get a takeaway from South Asian restaurant…* {Focus Group 3; Participant 2, Pakistan}

#### Sub-theme 4·2: Time pressures and shift towards convenience foods

Forming a continuity of sub-theme 1·5, participants felt that the busy lifestyle in Australia forced them to compromise and forego more complex traditional dishes and even substitute ingredients for time efficiency. Also, rather than cooking daily which was the norm in their previous home countries, participants reported preparing meals a few days in advance and reusing them or opting for frozen pre-prepared meals. Personal preferences in terms of mood and taste also reportedly influenced food choices coupled with lifestyle choices.

*…when we moved [to] Australia…our diet has completely changed…with this, [and] the busy schedule…everybody is working and then we have no house helps, you know, like [in] India, Pakistan…our traditional foods take a lot of time in cooking…I don’t have that much time.* {Focus group 4; Participant 4, India}

*…substituting a lot of what would have taken some time to make with ready- made…frozen meals from within the supermarkets, and then essentially introduce…a lot of…frozen items as well due to…accessibility and ease.* {Focus Group 1; Participant 3, India}

### Theme 5: Shifting perceptions around healthy eating

Participants discussed the influence of accessibility to cooking equipment (e.g. air fryers) and ingredients (seasonal availability) on eating practices. Further, they described how information and knowledge (e.g. food labels) have informed their food choices and portions. In essence, the theme reflected participants’ growing awareness and efforts to adjust not what they ate, but how they prepared and portioned it

#### Sub-theme 5·1: Conscious dietary modifications

Participants identified that certain changes to cooking were adopted to make foods healthier. These included ingredient substitutions such as swapping ghee with olive oil, changing cooking methods (steaming *v* frying) and adding more vegetables to recipes.

*…one thing that has changed is that whenever my mum would cook she before she would use canola oil, but now…because of Western influence she’s trying to like cook more healthier, even if she makes traditional dishes, so she’d opt for olive oil or something instead of Canola oil…so just choosing alternatives that are more healthy but don’t really impact the traditional dishes that much.* {Focus Group 3; Participant 3, India}

#### Sub-theme 5·2: Mindful eating

Participants recognised the shift seen amongst them and their families towards being health conscious in food choices. These extended beyond meals to conscious selection of ingredients, and recipes owing to food labels, greater health literacy and overall nutritional knowledge.

*…I’ve been more aware of the food contents. I do tend to look at the product descriptions. I do tend to look at product health ratings. If they’re healthier if they’re not.* {Focus Group 3; Participant 2, Pakistan}

#### Sub-theme 5·3: Mindful plating

Participants recognised that portion control is imperative when eating healthily highlighting a need for compromise between traditional meals and portion sizes for a healthy balance.

*…it would just come down to…how you portion things. So, instead of…having…rice like 70 % of your meal being rice, you can just change that to…70 % of like veggies or like curries…reducing the amount of rice.* {Focus Group 1; Participant 2, Pakistan}

### Theme 6: Empowerment through knowledge

While not central to acculturation particularly, participants identified the power of knowledge emphasising that information access and health awareness empowered them to adapt and make informed choices, thus influencing acculturative outcomes. Inclusive education targeting both young and older adults who form their network (family and friends) was also highlighted as the key supportive mechanism influencing dietary behaviours.

#### Sub-theme 6·1: Digital platforms as enablers

Participants identified the benefits of digital platforms in promoting healthy eating outlining the role of apps and social media to access new recipes, and aid self-monitoring.

*…a resource that could help ensure a healthy diet…could be like one of those calorie tracking apps that help you track how much calories ’you’re eating on a daily basis. So, if ’you’re on a caloric deficit, or you just trying to maintain yourself. Then you sort of have an idea of how much you’re eating so you’re not overeating or undereating.* {Focus Group 3; Participant 3, India}

#### Sub-theme 6·2: Impact of food labels and health policy

Some participants acknowledged the role of health policy in shaping eating behaviours among this group in Australia. Particularly, policies related to sugar consumption and nutrition labelling were recognised as being supportive for food literacy.

*…generally, just campaigning with the restaurants and the hospitality industry to enforce…that the restaurants are serving enough balanced diet in their meals and in their menus generally. So, in this way the message gets delivered to the kids, the homemakers, and…to people who are buying generally food from outside. So, I think it encompasses the whole society, overall.* {Focus Group 3, Participant 2, Pakistan}

#### Sub-theme 6·3: Navigating health knowledge in a multicultural context

Participants noted a need for having individualised, culturally sensitive education guidance targeted at both young adults and their families who influence their food choices. Participants suggested accessible platforms (such as schools, online platforms and healthcare) and advocated for adapting messages to relate across generations. The influence of Western and international centric dietary patterns such as the ‘Mediterranean diet’ was seen as limiting, with many asserting that ‘South Asian diets could be healthy with proper guidance on portion control’.

*…social media is…good for people our age…but I know…my parents aren’t on social media…even like educating them on what a healthy diet looks like, and what it contains…cause my parents do more of the cooking…I’m sure targeting the younger generations…be better because we’re gonna get older.* {Focus Group 2, Participant 3, India}

## Discussion

To the authors’ knowledge, this is the first study examining the drivers of dietary behaviours of young SAM in Australia. The findings reveal that acculturation, access to diverse food cultures and adaptation to the Australian food environment significantly influence dietary behaviours among young SAM. While cultural continuity remains strong in family settings and religious observance, acculturative adaptation is evident in cooking styles, external food choices and the use of digital technologies. Importantly, dietary behaviours were shaped by a dynamic interaction of socio-cultural norms, environmental exposures, health literacy and practical limitations, aligning with the study’s aims to explore drivers of dietary behaviours through the lens of acculturation. The study’s findings highlight a complex interplay of multiple factors influencing dietary behaviours in young SAM, illustrating how acculturation intersects with everyday dietary practices. Six key themes emerged as key drivers extending beyond acculturation, highlighting different dimensions of adaptation and continuity in participants’ food choices, providing greater insights into the dietary behaviours among young SAM.

The theme of ‘acculturation and exposure to diverse cuisines’ (Theme 1) reflects Australia’s multicultural food environment in shaping dietary practices. Participants reported both incorporating new cuisines and retaining South Asian food traditions. These findings align with the concept of bicultural acculturation, where individuals maintain cultural identity while adopting host country influences^(34)^. This challenges unidirectional models of dietary acculturation that assumes a linear shift towards Western diets and supports frameworks which account for bidirectional assimilation^(34)^. Particularly, the presence of culturally specific food outlets may suggest elements of bidirectional assimilation, where the host environment adapts to meet the cultural needs of migrant communities, potentially explaining why religious and cultural restrictions were not prominently discussed in focus groups. The blending of traditional and host country practices was also evident within this theme. Participants substituted ingredients, adopted new cooking techniques and integrated South Asian flavours into Western food preparation. These adaptations were not seen as compromises but as practical, health-conscious and new identity building strategies. This aligns with the findings from other migrant groups, such as Chinese, where food practices are used to negotiate identity, belonging and health^(35)^.

Family influence emerged as a key driver in the study, cutting across multiple themes, with participants emphasising the importance of familial dynamics in food choices. Intra-family relations, whether living with parents or independently, many retained strong food connections through home and takeaway food choices, including food prepared for special occasions. Notably, those living with family were more likely to report consuming traditional foods, while independent participants described greater autonomy but also challenges around time and preparation, often favouring convenience of Western meals. This interplay of living arrangements and family roles has been noted in other studies highlighting the intergenerational transmission of dietary norms^(36,37)^. For participants, focus group discussions revealed the need to manage inherited food practices alongside peer and cultural influences highlighting the importance of generational context in acculturation research.

For SAM, the role of religion in dietary behaviours was a salient theme in this study, often intertwined with cultural identity. Religious practices, such as fasting during specific periods like Ramadan (Islam) or Ekadashi (Hinduism), and cultural celebrations, such as Diwali and Eid, play a crucial role in maintaining cooking traditions and influencing food choices. While this theme captures the influence of religion and culture predominantly through special events and festive occasions, there appears to be a limited reflection of everyday religious practices in participants’ routine food choices, shopping habits or food preparation. For example, despite participants mentioning that many food outlets in Western Sydney are halal (Subtheme 1·5), there is little elaboration on how religious food rules shape decisions in cooking and shopping. Instead, food choices in everyday contexts were more often described in terms of convenience, time efficiency or health trends (Themes 1, 3 and 5). This raises an interesting point that religious dietary principles may be more salient during specific events rather than daily practice or it may be part of routine decisions and considered as the norm. This aspect needs further exploration to understand whether these religious practices are being quietly maintained and not verbalised or whether they are fading in importance of daily life. Or they could be so embedded that participants do not feel the need to mention them explicitly when discussing daily habits. Although cultural influences have been noted in previous research^(38,39)^, the influence of religion on dietary practices was not recognised. The religious continuity despite broader acculturation suggests that faith-based dietary practices remain strong anchors in navigating food environments in diaspora contexts. Understanding these practices is vital for health professionals delivering culturally sensitive dietary counselling or designing community programs.

The complexities of decision-making processes driven by social, economic and lifestyle considerations were emphasised by the themes 3 and 4. Participants identified social pressure, financial restraints and lifestyle demands as important barriers to adopting healthier eating habits. Similar findings are identified in other studies that highlight the influence of economic drivers on food choice, such as the higher cost of fruits and vegetables in Australia compared with their home countries^(38)^ and the impact of inflation and the increased cost of living, where cheaper, unhealthy food items are often chosen over healthier alternatives. However, it must be noted that the participants within this study are young adults predominantly living with family and these stressors may be different to people who follow various other migration streams which requires further exploration. Participants identified being family-oriented, valuing respect for elders and care for the young, which is derived from their expression of feeding family and friends’ home-cooked meals and eating large portions at community gatherings to show gratitude. However, negative judgement and social pressures hinder healthy lifestyle changes, as individuals may feel insecure or uncomfortable when attempting to diet or eat healthily, even in social settings such as dining out^(40)^. This reflects broader sociocultural norms around food and hospitality in South Asian communities where food is both a social incentive and cultural expression, affecting behaviours and actions^(37,40)^.

Interestingly, while other studies have highlighted the role of stress, particularly due to acculturation, as a contributor to NCD among SAM^(41)^ this was not reported by participants in the current study, which may potentially be due to the use of focus groups rather than individual interviews or the relatively young, health-literate participant demographics in this study. Themes 5 (‘shifting perceptions around healthy eating’) and 6 (‘empowerment through knowledge’) indicate a growing health awareness among participants. Many described using digital platforms, food labels and portion control strategies to modify their diets while still preserving cultural identity. Such mindful eating practices influenced by nutrition knowledge and health policy reflect a degree of agency that contrasts with the view of migrants being passive adopters of Western food habits^(5)^. This individual agency alongside various dietary drivers within the group suggests that the participants’ healthy BMI may be a result of these health-conscious behavioural changes. However, it is important to note that SAM are at a higher risk of developing NCD such as T2D and cardiovascular disease (CVD) at a lower BMI suggesting the need for early interventions to change this trajectory^(42)^. Several participants further highlighted the significance of culturally tailored education, indicating the need for culturally appropriate interventions^(43)^. This highlights the need for inclusive public health communication that considers diverse food cultures and engages with communities in the design of health promotion. Such targeted interventions may be beneficial in changing the BMI trajectory in this population especially with 63 % of SAM identified as either obese or overweight in Australia^(44)^.

The findings of this study affirm the significance of culturally responsive health promotion targets to bridge the health disparities in alignment with the National Preventive Health Strategy 2021–2030^(45)^. An effective public health response should ideally target the nuances of SAM dynamics specifically utilising a family-centric and culturally responsive approach by involving parents, elders and community leaders to improve effectiveness. Cultural and religious practices must be considered in dietary counselling, not just as barriers but as strengths. Digital platforms and peer-supported education may be effective tools for engaging young SAM by incorporating South Asian food cultures. Policy makers should prioritise strategies that address the structural and practical constraints faced by young SAM, such as time limitations and living alone. Ensuring the availability of healthy, convenient and culturally resonant food options may better support dietary choices than focussing solely on affordability and accessibility of cultural appropriateness.

### Strengths and limitations

One notable strength of this study is that this is the first study describing the drivers of food choices in young Australian SAM. While in-depth focus groups offered deeper insights, use of reflexivity and positionality by the researchers reduced interpretive bias. While these may have enhanced the transparency and trustworthiness of the findings, researchers’ familiarity with the culture may have led to some potential oversight. Some limitations identified include possible selection bias due to the method of sampling used in the study, which may have resulted in an overrepresentation of certain demographic groups. Although the eligibility criteria included individuals from across the South Asian region, the final sample was represented by 81 % of participants from India and Pakistan. This does not reflect the full cultural, linguistic, or religious diversity of South Asia, which includes countries such as Bhutan, Nepal and Maldives, each with distinct food cultures and traditions, thereby limiting generalisability. Additionally, the inclusion of both first- and second-generation SAM introduces heterogeneity, and this study did not stratify or analyse the responses separately. The sample also skewed towards those on a Halal diet due to an overrepresentation within a small sample of study participants, limiting generalisability. While the qualitative component of the study offers valuable insights, it lacks the ability to show causality or quantify connections between factors. Length of residence and breakdown of age groups were not collected, which would have limited assessment on dietary transition over time. Future studies would benefit from larger, stratified samples using mixed method approaches to better capture the diverse experiences of the broader SAM in Australia.

### Conclusions

This exploratory study offers valuable insights into the complex and multifaceted drivers of dietary behaviours among young SAM in Australia, highlighting how acculturation interacts with cultural identity, religion, family dynamics and socio-economic factors. Rather than viewing dietary behaviours solely through the overly simplified acculturation lens; the findings reveal a more nuanced and dynamic process, where participants selectively integrate elements of Western diets while maintaining cultural practices and traditions. Acculturation plays a role in dietary behaviours, although it is not the only factor influencing dietary changes or health disparities. Instead, dietary behaviours are shown to result from a web of interacting personal, structural and cultural forces, including food affordability, living arrangements, social norms and generational expectations. Familial influence emerged as a prominent driver, shaping both food choices and the emotional significance of food, especially during cultural and religious occasions. Recognising and supporting the bicultural dietary identity is key to promoting health equity and designing effective, inclusive nutrition interventions for Australia’s growing South Asian population.

Furthermore, the study underscores the rich diversity within the SAM community, characterised by varied interplays between different cultures and religions with familial influence emerging as a prominent theme. To achieve a meaningful reduction in the prevalence of NCD within these communities, public health policies and interventions must prioritise family dynamics and address intergenerational disparities. Future studies should consider exploring perspectives of older South Asian adults and strengthen evidence for family-centred interventions that consider cultural nuances and empower families to make healthier food choices. However, as the sample was not demographically representative of the broader South Asian region, this study should be considered as an early exploratory step in a larger body of work needed to understand the diverse experiences of SAM in Australia.

## Supporting information

Pawar et al. supplementary material Pawar et al. supplementary material

## Supplementary material

For supplementary material accompanying this paper visit https://doi.org/10.1017/S1368980025101250
