# Supplementary material for: Dietary behaviours of young South Asians in Australia: insights from a qualitative study
Source: Public Health Nutr. 2025 Oct 16;28(1):e178. doi: 10.1017/S1368980025101250 (PMC12722088; doi:10.1017/S1368980025101250)

**Supplement 1: Eligibility Screener**


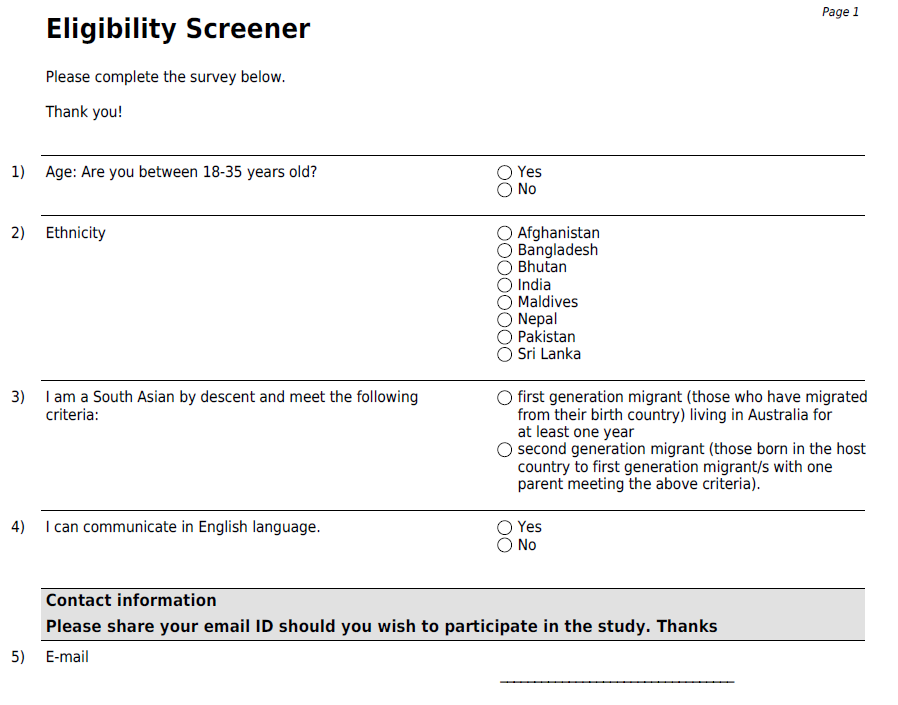


**Supplement 2: Semi-structured focus group guide**

Focus Group Guide for dietary behaviours study

Facilitators’ notes and schedule

| Welcome & introductions | Hello and welcome to the focus group session. Thanks for offering your time to participate in this session and share your insights and experience.  Today’ focus group will be conducted by two student dietitians, (introduce yourselves), Amani Fuad and Aboli Pawar. |
| --- | --- |
| Instructions to participants | You are part of this focus group because you volunteered to be part of the study and have signed the consent form. Please note that there are no correct or incorrect answers and all response matters.  As you would have noted in the participant information sheet whilst consenting to be part of this study, the focus group will be audio-recorded. The information collected however will be transcribed anonymously, which means none of you will be identifiable.  Please note that each of you can contribute by taking turns and listening when one person is speaking. The facilitators will ensure everyone gets an opportunity to speak and participate.  Please turn your mobile phones to silent mode if possible or reduce volume. If you need to take a phone call during the session, please do so by taking leave from the group. Please turn off your cameras now and mute your microphone unless when you are speaking. Please use raise your hand function on the zoom or verbally voice your intention to join in if you want to contribute at anytime or just voice something. The facilitators will offer each of your turns to have your say as well.  The session is expected to take approximately an hour to 1.5 hours. Shall we start the recording now? (start recording) |
| Key Message: | The aim of this focus group is to explore drivers of dietary behaviours. This information will provide deeper insights into the determinants of South Asian young adults’ food choices and dietary habits. Such information is valuable to understand its impact on health and wellbeing and may potentially guide policy and intervention targets to improve health outcomes for this group. |
| Participant introductions | Could we please start by self-introductions now? Please tell us your name and your dietary preferences (something like I follow a Halal diet, or I am a vegetarian, I prefer a Pescatarian diet due to personal choice, etc.). |
| Discussion topics | |
| 1 | 1. Now let’s each share what your typical meal looks like. So, what did you eat for dinner yesterday?   Probes if they are silent..  *E.g., Do you always have a set meal pattern, like 3 main meals? What will your typical lunch or dinner meal? And breakfast?*  b. I am sure there may be odd occasions which may change what and how you eat. Could you elaborate on specific occasions that comes to your mind?  *E.g., may be during cultural celebrations or religious events or on weekends?* |
| 2 | We would like to know how you have maintained your tradition here with dietary behaviours. (allow responses)  We are also interested to know how these have evolved being in Australia. How has exposure to Australian food culture influenced your dietary habits/food choices?  Probes: Explain whether maintaining traditions has been challenging for you. Do you find yourselves incorporating more non-traditional foods (Western, multi-cultural etc) into your diet? |
| 3 | Well, community can mean your new community or connections with your local South Asian community. Can you tell us how does your current community in Australia influence your dietary habits?  Probes: Do you tend to eat out often and if so, how does this look like? Do you rely on takeaway foods and what would these be? |
| 4 | We are interested to learn your understanding and perspectives on what healthy eating means within the context of South Asian cuisine or Australian food culture in general.  Discuss any barriers or challenges you encounter to eating healthy here in Australia. |
| 5 | Now, let’s discuss food preparation and cooking practices focusing on traditional cooking methods and their importance from a South Asian context. What are some traditional meals and cooking practices you are familiar of within your culture?  Great, do you feel these have preserved here after migration and if not, why?  If you are given a choice to opt for cooking your own meal; choosing a takeaway option from familiar South Asian menu or buying from a takeaway outlet serving Western or other non-South Asian meals, what would each of you opt for and why? |
| Close | Thank you so much for your valuable time and feedback. Before we close, is there anything you would like to add to this discussion? |

**Supplement 3: Short Demographics Form**


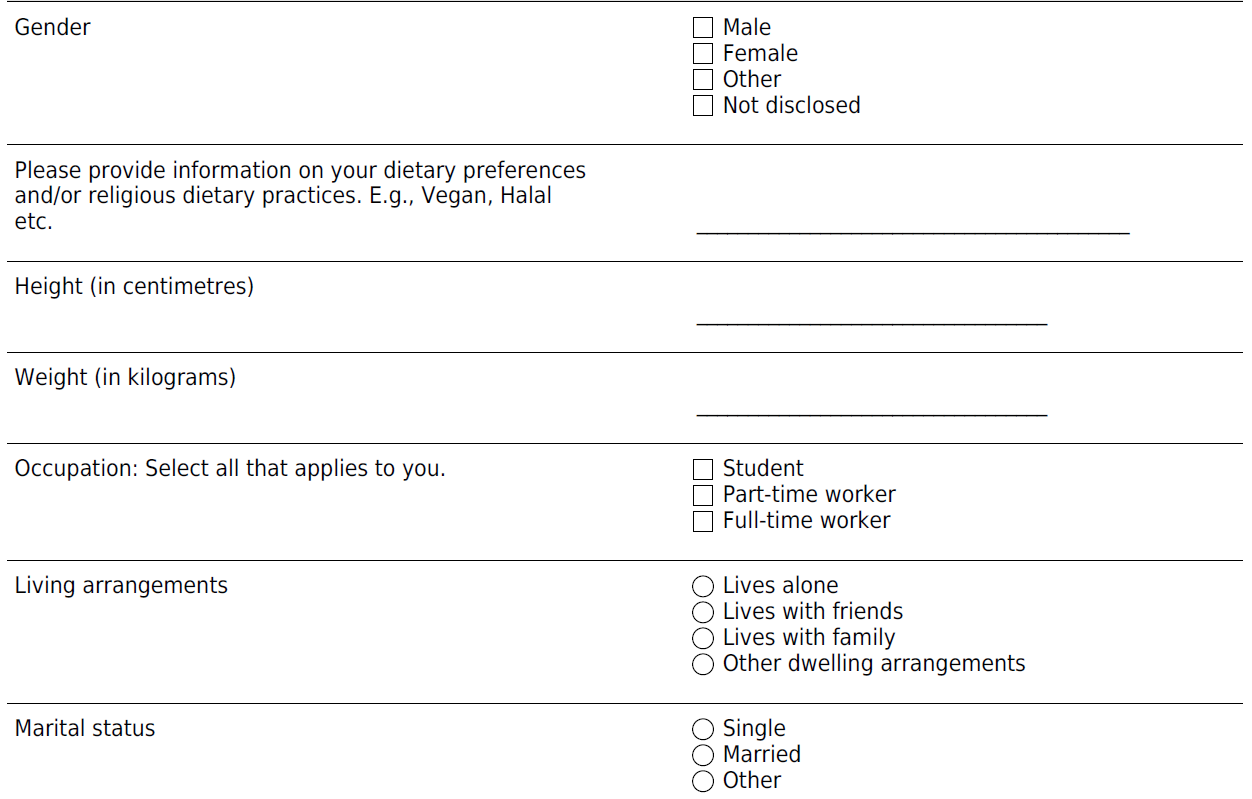

Supplement: Pawar et al. supplementary material [file S1368980025101250sup001.docx]
